# Association between body roundness index and frailty in chronic obstructive pulmonary disease: A cross-sectional study of NHANES 1999 to 2018

**DOI:** 10.1097/MD.0000000000046569

**Published:** 2025-12-12

**Authors:** Xiaohua Lin, Xiu He, Hongbo Xu, Zhijun Suo, Yunsheng Yuan

**Affiliations:** a Department of Critical Care Medicine, Shenzhen Nanshan People’s Hospital, Shenzhen, China.

**Keywords:** abdominal obesity, body roundness index, chronic obstructive pulmonary disease, frailty, NHANES

## Abstract

Frailty in chronic obstructive pulmonary disease (COPD) is linked to adverse outcomes, with emerging evidence implicating abdominal obesity. This study examines the association between the body roundness index (BRI), a novel measure of body fat distribution, and frailty in COPD patients. Using cross-sectional data from the National Health and Nutrition Examination Survey (NHANES, 1999–2018), 1151 adults ≥ 20 years with COPD were analyzed. Frailty was defined as a frailty index ≥ 0.21. BRI, calculated from waist circumference and height, was evaluated as continuous and tertiles. Adjusted weighted logistic regression and restricted cubic spline models assessed associations, controlling for sociodemographic, lifestyle, and clinical factors. Higher BRI was independently associated with increased frailty risk. Each unit rise in BRI elevated frailty odds by 14% (OR: 1.14, 95% CI: 1.04–1.24) in fully adjusted models. The risk of frailty was significantly increased in the highest BRI tertile compared to the lowest BRI tertile (OR: 1.95, 95% CI: 1.21–3.13, *P* = .01). Subgroup analyses showed a consistent positive association between BRI and frailty. Elevated BRI, reflecting central obesity, is strongly linked to frailty in COPD. Targeting visceral fat reduction may mitigate frailty progression. Longitudinal studies are needed to confirm causality.

## 1. Introduction

Chronic obstructive pulmonary disease (COPD) is a leading cause of morbidity and mortality worldwide, disproportionately affecting older adults.^[1]^ The progressive decline in lung function characteristic of COPD often leads to reduced functional capacity and overall health, significantly impacting patients’ quality of life.^[2–4]^ Complicating this challenge is the increasing recognition of frailty as a critical determinant of COPD severity and progression. Frailty, defined as a state of diminished physiological reserve and increased vulnerability to stressors, is strongly associated with adverse outcomes in COPD, including accelerated disease progression, increased symptom burden, higher hospitalization rates and increased mortality risk.^[5–7]^

Although frailty has traditionally been associated with weight loss and malnutrition, recent studies have highlighted the important role of obesity, particularly abdominal obesity, in the development of frailty.^[3,8]^ This paradigm shift emphasizes the importance of considering body fat distribution rather than relying solely on body weight or body mass index (BMI) when assessing frailty risk. BMI is a widely used measure of obesity, but its inability to capture nuances in body composition can be misleading, especially in older adults who experience age-related changes in muscle mass and fat distribution.^[9,10]^ Abdominal obesity, commonly measured by waist circumference (WC), is a better predictor of frailty than BMI, possibly because abdominal obesity is associated with metabolic dysfunction, including insulin resistance and chronic inflammation, both of which are implicated in the pathogenesis of frailty.^[8,11]^

Compared to BMI, body roundness index (BRI) is a novel anthropometric measure that combines WC and height to provide a more comprehensive and accurate assessment of body fat and visceral fat content.^[10,12]^ Importantly, the BRI can identify individuals with abdominal obesity who may not be classified as obese based on BMI alone, particularly those with normal body weight but a high percentage of visceral fat.^[9]^ Studies have shown that BRI is associated with a variety of metabolic disorders, including diabetes, cardiovascular disease, and metabolic syndrome, all of which share pathophysiological pathways with frailty.^[10,13–16]^ Despite the growing body of evidence supporting the association of BRI with metabolic health, its relationship with frailty in patients with COPD remains largely unexplored.

Because of the high prevalence of both abdominal obesity and frailty in patients with COPD, understanding the role of body fat distribution in the development of frailty in patients with COPD may help to develop targeted interventions to prevent or mitigate frailty and improve the overall health of this vulnerable population. Therefore, the aim of this study was to elucidate the relationship between BRI and frailty in patients with COPD through a cross-sectional analysis of data from the National Health and Nutrition Examination Survey (NHANES) from 1999 to 2018.

## 2. Materials and methods

### 2.1. Source of data and study population

This study utilizes data from the NHANES, a nationally representative, cross-sectional survey conducted by the Centers for Disease Control and Prevention (CDC) to assess the health and nutritional status of the noninstitutionalized civilian population of the United States. NHANES employs a complex, multistage, probability sampling design and collects data through in-home interviews and physical examinations at a mobile examination center (MEC). The interviews gather demographic, socioeconomic, dietary, and health-related information, while the MEC examinations, conducted by trained medical personnel, include medical, dental, and physiological measurements, as well as laboratory tests. All NHANES survey protocols are approved by the NCHS Research Ethics Review Board at the CDC, and all participants provide written informed consent. As this study uses de-identified, publicly available data from NHANES, it is exempt from further ethical review and informed consent.

**Figure 1.**
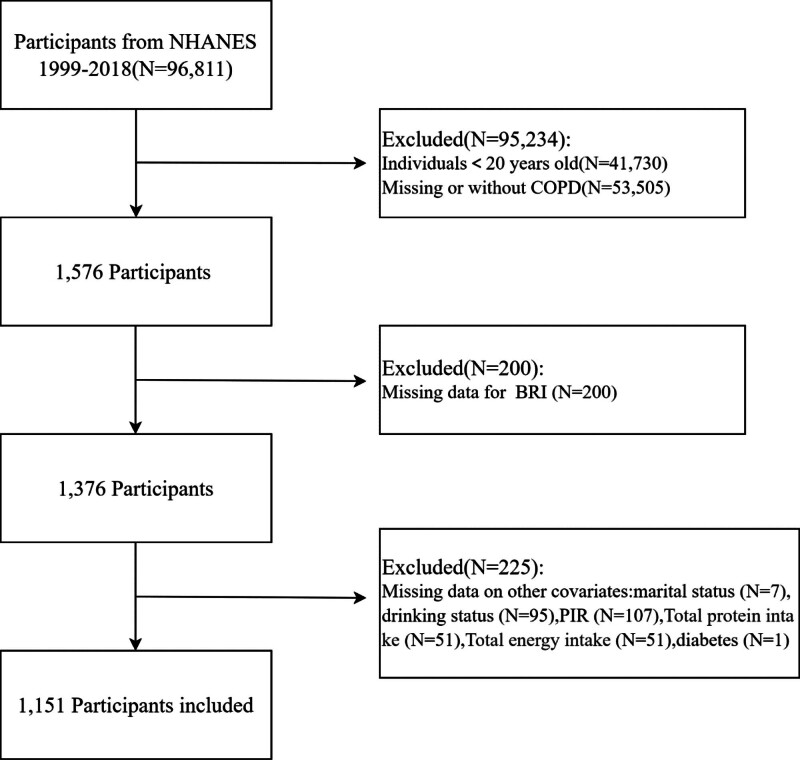
Flow chart for inclusion and exclusion in this study. NHANES = National Health and Nutrition Examination Survey.

Data from the NHANES from 1999 to 2018 was used for this study. COPD was defined as self-reporting a diagnosis of COPD or emphysema by a doctor or other health professional, or a postbronchodilator FEV1/FVC ratio < 0.70. The NHANES study from 1999 to 2018 initially identified 96,811 participants, 1576 participants with COPD were identified among adults aged ≥ 20 years. Exclusion criteria were as follows: participants missing BRI data (n = 200), and further exclusion of participants with missing covariate data including marital status (n = 7), drinking status (n = 95), poverty income ratio (PIR) (n = 107), total protein intake (n = 51), total energy intake (n = 51), and diabetes (n = 1). Ultimately, a total of 1151 participants were included in the present study (Fig. 1).

### 2.2. Definition of frailty

In this study, frailty was assessed using the frailty index (FI), a measure based on the accumulation of health deficits. The FI, originally developed by Rockwood and adapted to the NHANES dataset by Hakeem et al, is widely recognized as a robust tool for quantifying frailty.^[17,18]^ The FI includes 49 deficits across several physiological systems, including cognition, dependency, depression, comorbidities, hospitalization rates, general health perceptions, physical functioning, anthropometric measures, and laboratory results. Deficits within each system were determined based on participants’ self-reported responses to specific sections of the NHANES questionnaire. The FI is expressed as a ratio between 0 and 1, representing the proportion of observed deficits relative to the total number of potential deficits (Table S1, Supplemental Digital Content, https://links.lww.com/MD/Q915). An FI ≥ 0.21 was used to classify COPD patients as frail.^[19]^

### 2.3. Definition of BRI

The BRI is a novel anthropometric measure that provides a more accurate representation of body fat and visceral adipose tissue percentage than traditional indices such as BMI. The BRI is calculated using WC and height (BH) measurements collected from participants during physical examinations conducted at MEC as part of the NHANES. The formula for calculating is BRI = 364.2 − 365.5 × √[1 − (WC/(2π))^2^/(0.5 × BH)^2^].^[12,20]^

### 2.4. Covariate assessment

Covariates in this study included sociodemographic factors, lifestyle factors, and comorbidities. Sociodemographic factors encompassed age, gender, race, education level, and the family income-to-poverty level ratio (PIR). Marital status was categorized as either married or unmarried, with the unmarried group including individuals who were never married, separated, divorced, or widowed, and the married group including those who were married or living with a partner. The family income-to-poverty level ratio was further classified into 2 categories: <1.0 and equal to or >1.0.^[21]^

Lifestyle factors considered in the study included smoking status, alcohol consumption, total daily protein intake, and total energy intake. Smoking was divided into three categories: never smokers, ex-smokers, and current smokers. Alcohol consumption was classified into 5 groups: never drinkers, ex-drinkers, heavy drinkers, light drinkers, and moderate drinkers. BMI was categorized according to CDC guidelines as nonobese (<30) and obese (≥30).

The study also assessed the presence of comorbidities, specifically hypertension and diabetes. Hypertension was defined as having a mean systolic blood pressure ≥ 140 mm Hg, a mean diastolic blood pressure ≥ 90 mm Hg, self-reported hypertension, or being on antihypertensive medication.^[22]^ Diabetes was diagnosed based on several criteria, including the use of antihyperglycemic medication, an HbA1c concentration of ≥ 6.5%, a fasting plasma glucose level of ≥7.0 mmol/L, a 2-hour glucose level of ≥ 11.1 mmol/L during an oral glucose tolerance test, a random blood glucose level of ≥ 11.1 mmol/L, or self-reported diabetes.^[2]^

### 2.5. Statistical analysis

The study employed the complex multistage sampling design and sample weighting methodology utilized by NHANES. All analyses were conducted using complete datasets without employing any estimation methods to address missing data. Categorical variables are presented as weighted percentages, while continuous variables are displayed as weighted means with standard errors. To compare characteristics across BRI groups (tertiles), chi-square tests were performed for categorical variables and Kruskal–Wallis tests for non-normally distributed continuous variables. Weighted multiple logistic regression analyses were conducted, adjusting for different covariates by treating BRI both as a continuous variable and as a categorical variable in separate models.

Model 1: unadjusted.

Model 2: adjusted for race, age, gender, education level, PIR, and marital status.

Model 3: further adjusted for smoking status, alcohol consumption, hypertension, and diabetes, in addition to the variables in Model 2.

Additionally, Model 3 utilized weighted restricted cubic spline (RCS) curves to evaluate the relationship between BRI and frailty. Subgroup analyses were performed based on age, gender, race, education level, PIR, smoking status, marital status, drinking status, hypertension, and diabetes.

To investigate the long-term prognostic value of the BRI on patient outcomes, a survival analysis was conducted using all-cause mortality as the primary endpoint. Mortality data were obtained from the linked mortality files of the NHANES. The follow-up period was calculated from the date of the baseline interview to the date of death or the end of the follow-up period (December 31, 2018), whichever came first. The Kaplan–Meier method was used to generate survival curves for each BRI tertile, and the log-rank test was employed to compare the survival distributions among the groups.

Statistical analyses were conducted using R version 4.4.1 and R packages (R Foundation for Statistical Computing, Vienna, Austria) (e.g., “survey,” “rms,” “ggplot”). Two-sided *P*-values of <.05 were considered statistically significant.

## 3. Results

### 3.1. Baseline characteristics

Baseline characteristics of 1151 participants with COPD were compared according to tertiles of BRI, which was divided into 3 groups: Q1 (1.472, 4.455), Q2 (4.455, 6.292), and Q3 (6.292, 17.078). As shown in Table 1, the median age was 30 years, with 57.37% male, 42.63% female, and 576 participants classified as frail. Participants in the highest BRI tertile (Q3) were older, had a higher BMI, and showed a greater prevalence of hypertension, diabetes, and frailty. Furthermore, comparisons based on frailty status revealed that frail participants were older, had a higher BMI, and exhibited higher proportions of smoking and alcohol consumption, while their daily energy and protein intake, as well as income levels, were lower (Table S2, Supplemental Digital Content, https://links.lww.com/MD/Q915).

**Table 1:** Baseline characteristics of participants according to tertiles of BRI.

Characteristics	Total (N = 1151)	Q1 (1.472, 4.455) (N = 384)	Q2 (4.455, 6.292) (N = 384)	Q3 (6.292, 17.078) (N = 383)	*P*-value
Age, yr	61 (53,71)	58 (48,69)	63 (53,71)	64 (56,72)	<.0001
Gender (%)					
Female	416 (42.63)	136 (44.46)	120 (39.83)	160 (43.67)	.56
Male	735 (57.37)	248 (55.54)	264 (60.17)	223 (56.33)	
Race/ethnicity (%)					
Mexican American	56 (1.21)	9 (0.62)	20 (1.29)	27 (1.73)	.27
Non-Hispanic Black	168 (5.95)	74 (7.64)	46 (4.82)	48 (5.40)	
Non-Hispanic White	807 (84.80)	269 (85.01)	274 (84.09)	264 (85.34)	
Other Hispanic	57 (1.91)	12 (0.98)	19 (2.72)	26 (2.03)	
Other Race	63 (6.12)	20 (5.75)	25 (7.08)	18 (5.50)	
Marital status (%)					
Married	651 (60.98)	212 (58.03)	239 (66.47)	200 (58.28)	.1
Nonmarried	500 (39.02)	172 (41.97)	145 (33.53)	183 (41.72)	
PIR (%)					
< 1	271 (17.22)	104 (19.85)	77 (13.17)	90 (18.75)	.07
≥1	880 (82.78)	280 (80.15)	307 (86.83)	293 (81.25)	
Educational level (%)					
College graduate or above	104 (10.36)	40 (12.85)	36 (11.12)	28 (6.98)	.23
High school or below	765 (63.46)	255 (60.18)	251 (64.71)	259 (65.53)	
Some college or associated degree	282 (26.19)	89 (26.97)	97 (24.17)	96 (27.49)	
Smoking (%)					
Current	480 (42.96)	216 (57.67)	138 (38.81)	126 (32.07)	<.0001
Former	514 (42.85)	112 (25.62)	195 (48.61)	207 (54.65)	
Never	157 (14.19)	56 (16.71)	51 (12.58)	50 (13.28)	
Drinking (%)					
Former	371 (28.13)	109 (24.54)	116 (25.86)	146 (34.24)	.02
Heavy	187 (17.69)	81 (22.39)	57 (15.98)	49 (14.63)	
Mild	383 (33.52)	116 (28.60)	147 (39.13)	120 (32.75)	
Moderate	140 (15.16)	56 (19.60)	46 (14.82)	38 (10.91)	
Never	70 (5.49)	22 (4.87)	18 (4.22)	30 (7.48)	
Energy intake (kcal/d)	1851 (1369,2475)	1972 (1491,2556)	1774.64 (1337,2422)	1817 (1280,2475)	.06
Protein intake (g/d)	69.69 (47.84, 94.17)	70.89 (47.90, 95.52)	69.44 (48.35, 91.94)	67.91 (47.83, 96.26)	.91
BMI (kg m^2^)	27.00 (23.70, 31.63)	22.28 (20.09, 24.13)	27.10 (25.60, 29.04)	33.80 (31.13, 38.30)	<.0001
Hypertension (%)					
No	444 (43.29)	207 (58.64)	135 (43.84)	102 (26.83)	<.0001
Yes	707 (56.71)	177 (41.36)	249 (56.16)	281 (73.17)	
DM (%)					
No	851 (78.48)	348 (93.96)	298 (83.11)	205 (57.62)	<.0001
Yes	300 (21.52)	36 (6.04)	86 (16.89)	178 (42.38)	
Frailty (%)					
No	575 (55.40)	236 (66.90)	206 (60.09)	133 (38.57)	<.0001
Yes	576 (44.60)	148 (33.10)	178 (39.91)	250 (61.43)	

BMI = body mass index, DM = diabetes, PIR = family income-to-poverty level ratio.

### 3.2. Association between BRI and frailty risk

The association between BRI and frailty among COPD patients was investigated using weighted multivariable logistic regression analysis. As shown in Table 2, when BRI was analyzed as a continuous variable, the odds ratios (ORs) in models 1 to 3 were 1.28 (95% CI: 1.17–1.39), 1.28 (1.17, 1.40), and 1.14 (1.04, 1.24), respectively. The fully adjusted model indicated that each unit increase in BRI was associated with a 14% higher risk of frailty. Moreover, when BRI was categorized into tertiles, model 3 (adjusted for age, sex, race, education, marital status, PIR, smoking, drinking, hypertension, and diabetes) demonstrated a significantly increased risk of frailty in the highest tertile of the BRI compared to the lowest tertile (OR [95% CI]: 1.95 [1.21, 3.13], *P*-value = .01). In all models, there was a significant trend of increasing frailty risk with increasing BRI tertiles (*P* for trend < .05).

**Table 2:** Associations of BRI with frailty among patients with COPD.

BRI	Model 1		Model 2		Model 3	
	OR (95% CI)	*P*-value	OR (95% CI)	*P*-value	OR (95% CI)	*P*-value
**Continuous** Per 1 unit increase **Tertiles**	1.28 (1.17, 1.39)	<.001	1.28 (1.17, 1.40)	<.001	1.14 (1.04, 1.24)	.01
Q1	Ref		Ref		Ref	
Q2	1.34 (0.89, 2.01)	.15	1.49 (0.96, 2.30)	.07	1.21 (0.76, 1.94)	.42
Q3	3.22 (2.09, 4.96)	<.001	3.35 (2.11, 5.32)	<.001	1.95 (1.21, 3.13)	.01
*P* for trend		<.001		<.001		.002

Model 1: unadjusted.

Model 2: adjusted for race, age, gender, education level, PIR, and marital status.

Model 3: further adjusted for smoking status, alcohol consumption, hypertension, and diabetes, in addition to the variables in model 2.

The BRI converted to a categorical variable (tertiles) is consistent with Table 1.

BRI = body roundness index, CI = confidence interval, OR = odds ratio.

### 3.3. Subgroup and RCS analyses

**Figure 2.**
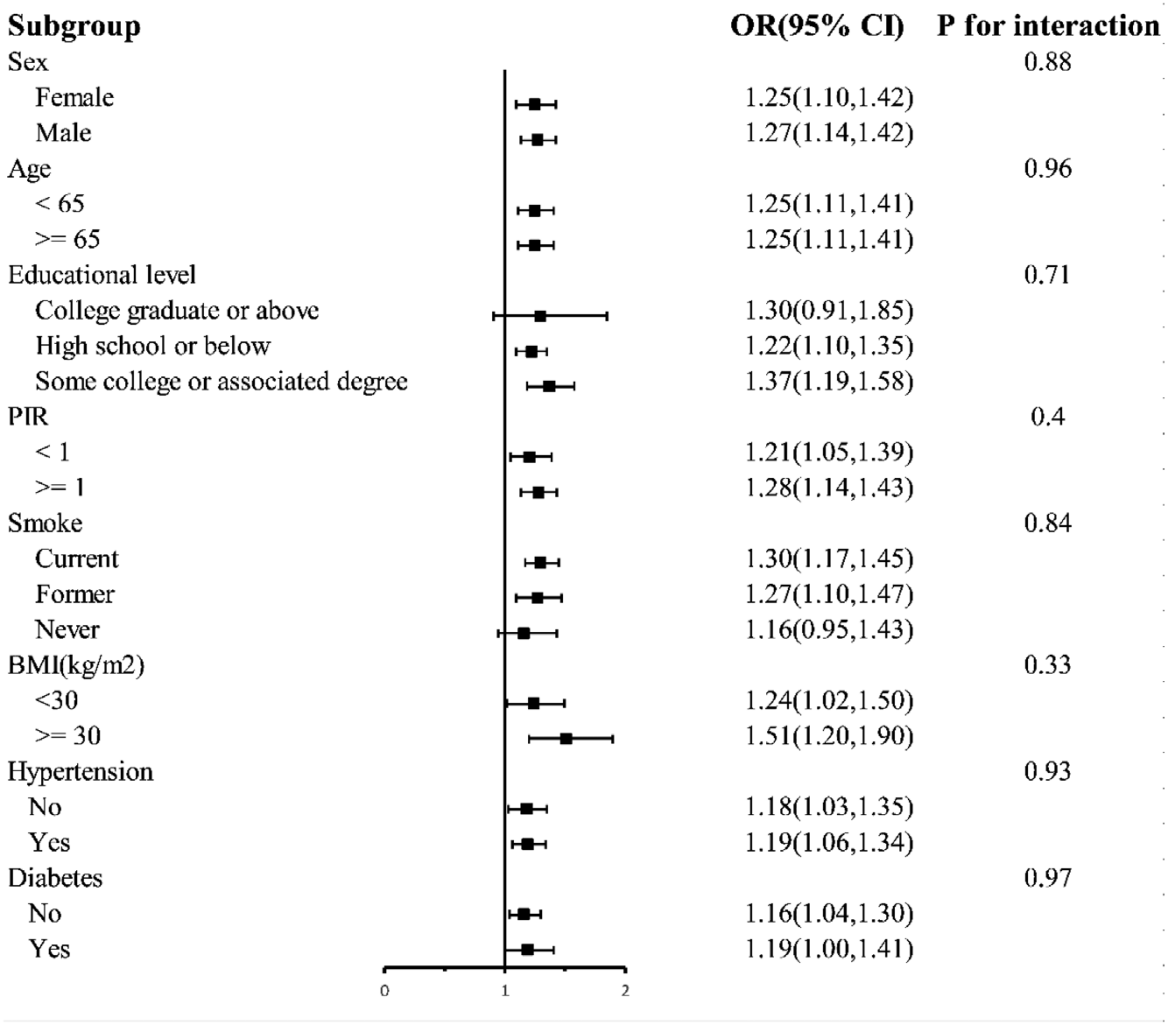
Association between BRI and frailty across subgroups. Adjusted for age, sex, race, education, marital status, PIR, smoking, drinking, hypertension, and diabetes. BRI = body roundness index, CI = confidence interval, OR = odds ratio, PIR = poverty income ratio.

**Figure 3.**
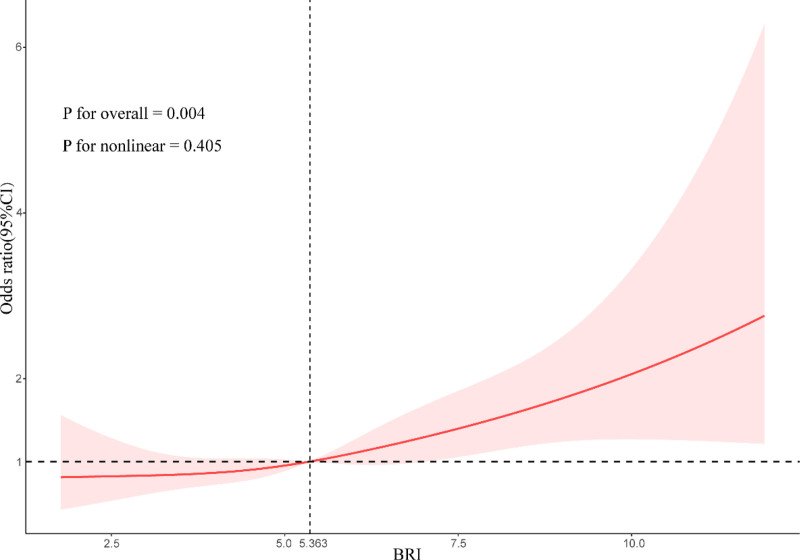
Restricted cubic spline fitting for the association between BRI and frailty adjusted for age, sex, race, education, marital status, PIR, smoking, drinking, hypertension, and diabetes. BRI = body roundness index, CI = confidence interval, PIR = poverty income ratio.

Subgroup analyses were performed to further explore the association between BRI and frailty. Stratification by age, sex, education level, PIR, smoking, BMI, hypertension, and diabetes revealed a positive association between BRI and frailty, with no significant interactions (Fig. 2). RCS analysis showed that at a BRI of 5.363 or higher, there was an overall trend toward a significantly increased risk of frailty (*P* overall = .004), but no significant nonlinear relationship between BRI and frailty was observed (*P*nonlinear = .405)(Fig. 3).

### 3.4. Kaplan–Meier survival analysis

**Figure 4.**
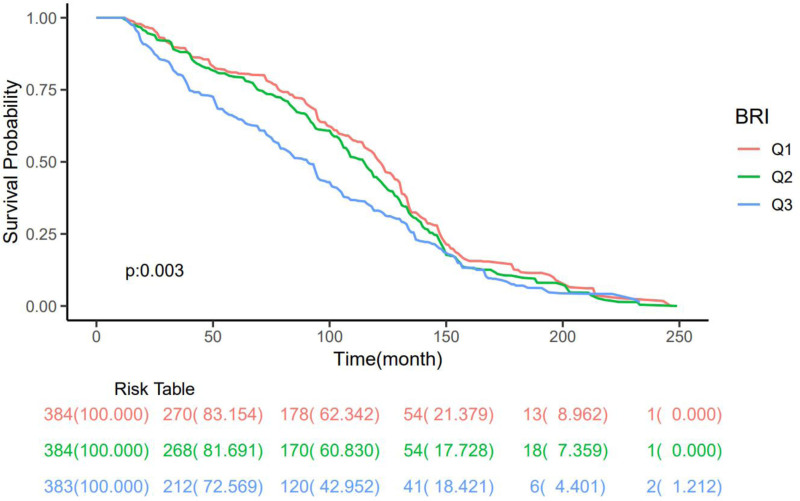
Kaplan–Meier survival curves for all-cause mortality by BRI tertiles. Patients were stratified into 3 groups: Q1 (lowest BRI), Q2 (middle BRI), and Q3 (highest BRI). The *P*-value was derived from the log-rank test. The risk table below the plot indicates the number of patients at risk at specified time intervals. BRI = body roundness index.

The Kaplan–Meier survival analysis revealed a significant difference in all-cause mortality across the BRI tertiles (Fig. 4). The survival curve for patients in the highest BRI tertile (Q3) was visibly separated from and consistently lower than the curves for the Q1 (lowest BRI) and Q2 (middle BRI) tertiles, which largely overlapped. The log-rank test confirmed that this difference in survival distributions among the 3 groups was highly statistically significant (*P* = .003).

## 4. Discussion

This study examined the association between BRI and frailty in COPD. In patients with COPD, BRI was significantly and positively associated with the risk of frailty, and this association persisted after adjustment for multiple confounders, i.e., the higher the BRI, the greater the risk of frailty.

BRI is an indicator of central obesity and also reflects the accumulation of visceral fat. Visceral adipose tissue secretes a variety of pro-inflammatory factors such as IL-6 and TNF-α, leading to a chronic low-grade inflammatory state in the body.^[4,23]^ Chronic inflammation is one of the important pathophysiological mechanisms for the development of frailty.^[24]^ COPD is a chronic inflammatory disease,^[23]^ and the inflammatory state exacerbated by central obesity may further contribute to the development of frailty in COPD patients.^[3,25]^ In addition, central obesity is strongly associated with insulin resistance, which has been shown to be associated with an increased risk of frailty.^[20,26]^ Insulin resistance may increase the risk of frailty by affecting the balance between muscle protein synthesis and catabolism, resulting in decreased muscle mass and strength.^[27]^ Obesity also leads to a decrease in muscle mass and strength, which are key features of frailty.^[28,29]^ Our results suggested that higher BRI may be an important risk factor for the development of frailty in COPD patients. This is consistent with previous studies that obesity, especially central obesity, is associated with an increased risk of frailty.^[9,30,31]^

In addition, the subgroup analyses performed in this study showed that in obese patients, the risk of frailty was significantly increased with increasing BRI. Similarly, in COPD patients, after full adjustment for confounding variables, the risk of frailty was significantly increased when the BRI was higher than 5.363. These findings highlight the potential of the BRI as an important indicator for assessing the risk of frailty in patients with COPD. In clinical practice, healthcare providers can use BRI to screen COPD patients for frailty and provide early intervention to control body weight and reduce BRI in high-risk groups, especially obesity, which may help reduce the risk of frailty and improve the prognosis of patients.

The main strengths of this study are the first confirmation of the positive association between BRI and frailty in COPD patients, followed by the collection of large sample data from the NHANES database, which enhances the generalizability of the findings. In addition, this study used a variety of statistical methods, including weighted multivariate logistic regression modeling, subgroup analysis, and RCS modeling, to comprehensively and thoroughly explore the relationship between BRI and frailty and to verify the robustness of the results. However, this study has several limitations. First, this is a cross-sectional study, and the causal relationship between BRI and frailty could not be determined. Second, the frailty assessment method we used was based on a self-reported questionnaire, which may be subject to recall bias. Third, the population in this study was mainly from the NHANES database, and its representativeness may be somewhat limited. Future studies are needed to validate our findings in different populations.

## 5. Conclusion

In patients with COPD, increased BRI is significantly associated with an increased risk of frailty. Nutritional management based on BRI, especially focusing on reducing central obesity, is an important strategy to prevent and delay the development of frailty in COPD patients. Future studies are needed to validate this finding and explore its mechanisms in order to develop targeted interventions to reduce the risk of frailty in COPD patients.

## Author contributions

**Conceptualization:** Zhijun Suo.

**Formal analysis:** Xiu He, Hongbo Xu.

**Investigation:** Xiu He, Hongbo Xu

**Methodology:** Xiaohua Lin, Xiu He, Hongbo Xu, Zhijun Suo.

**Supervision:** Zhijun Suo.

**Validation:** Xiaohua Lin, Hongbo Xu, Zhijun Suo, Yunsheng Yuan.

**Writing – original draft:** Xiaohua Lin.

**Writing – review & editing:** Yunsheng Yuan.

## Supplementary Material
