# Supplementary material for: Association between body roundness index and frailty in chronic obstructive pulmonary disease: A cross-sectional study of NHANES 1999 to 2018
Source: Medicine (Baltimore). 2025 Dec 12;104(50):e46569. doi: 10.1097/MD.0000000000046569 (PMC12708219; doi:10.1097/MD.0000000000046569)
Supplement: Supplementary file 1 [file medi-104-e46569-s001.docx]

**Table S1:**Variables in the 49-Item Frailty Index and Their Respective Scorings[1]

| Variable | Scoring | |  |
| --- | --- | --- | --- |
| COGNITION | | | |
| 1. Experience confusion/memory problems | Yes=1, No=0 | |  |
| DEPENDENCE | | | |
| 2. Managing money | Difficulty=1, No Difficulty=0 | |  |
| 3. Stooping, crouching, kneeling | Difficulty=1, No Difficulty=0 | |  |
| 4. Lifting or carrying | Difficulty=1, No Difficulty=0 | |  |
| 5. House chore | Difficulty=1, No Difficulty=0 | |  |
| 6. Preparing meals | Difficulty=1, No Difficulty=0 | |  |
| 7. Standing up from armless chair | Difficulty=1, No Difficulty=0 | |  |
| 8. Getting in and out of bed difficulty | Difficulty=1, No Difficulty=0 | |  |
| 9. Using fork, knife, drinking from cup | Difficulty=1, No Difficulty=0 | |  |
| 10. Dressing yourself | Difficulty=1, No Difficulty=0 | |  |
| 11. Standing for long periods difficulty | Difficulty=1, No Difficulty=0 | |  |
| 12. Grasp/holding small objects | Difficulty=1, No Difficulty=0 | |  |
| 13. Attending social event | Difficulty=1, No Difficulty=0 | |  |
| 14. Push or pull large objects | Difficulty=1, No Difficulty=0 | |  |
| 15. Walking for a quarter mile difficulty | Difficulty=1, No Difficulty=0 | |  |
| 16. Walking up ten steps difficulty | Difficulty=1, No Difficulty=0 | |  |
| DEPRESSIVE SYMPTOMS | | | |
| 17. Have little interest in doing things | Nearly every day=1, More than half the days= 0.66, Several days= 0.33, Not at all=0 | |  |
| 18. Feeling down, depressed, or hopeless | Nearly every day=1, More than half the days= 0.66, Several days= 0.33, Not at all=0 | |  |
| 19. Trouble sleeping or sleeping too much | Nearly every day=1, More than half the days= 0.66, Several days= 0.33, Not at all=0 | |  |
| 20. Feeling tired or having little energy | Nearly every day=1, More than half the days= 0.66, Several days= 0.33, Not at all=0 | |  |
| 21. Poor appetite or overeating | Nearly every day=1, More than half the days= 0.66, Several days= 0.33, Not at all=0 | |  |
| 22. Feeling bad about yourself | Nearly every day=1, More than half the days= 0.66, Several days= 0.33, Not at all=0 | |  |
| 23. trouble concentrating on things | Nearly every day=1, More than half the days= 0.66, Several days= 0.33, Not at all=0 | |  |
| COMORBIDITIES | | | |
| 24. Arthritis | Yes=1, Suspect = 0.5 No=0 | |  |
| 25. Thyroid problems | Yes=1, Suspect = 0.5 No=0 | |  |
| 26. Chronic Bronchitis | Yes=1, Suspect = 0.5 No=0 | |  |
| 27. Cancer | Yes=1, Suspect = 0.5 No=0 | |  |
| 28. Congestive Heart Failure | Yes=1, Suspect = 0.5 No=0 | |  |
| 29. Coronary Heart Disease | Yes=1, Suspect = 0.5 No=0 | |  |
| 30. Angina | Yes=1, Suspect = 0.5 No=0 | |  |
| 31. Heart Attack | Yes=1, Suspect = 0.5 No=0 | |  |
| 32. Stroke | Yes=1, Suspect = 0.5 No=0 | |  |
| 33. Blood Pressure | Yes=1, Suspect = 0.5 No=0 | |  |
| 34. Diabetes | Yes=1, Suspect = 0.5 No=0 | |  |
| 35. weak/failing kidneys | Yes=1, Suspect = 0.5 No=0 | |  |
| 36. Urinary Leakage | Yes=1, Suspect = 0.5 No=0 | |  |
| Hospital Utilization & Access to Care | | | |
| 37. Self Rated Health | Fair, poor=1, Excellent, Very good, good=0 | |  |
| 38. Health now compared with 1 year ago | Worse=1, About the same, better =0 | |  |
| 39. Overnight hospital patient in last year | Yes=1, No=0 | |  |
| 40. Frequency of health care use during last year. | None=0, 1-5= 0,5, More than 5=1 | |  |
| 41. Number of Prescribed medications | None=0 , 1-4=0.5, Five and more= 1 | |  |
| PHYSICAL PERFORMANCE AND ANTHROPOMETRY | | | |
| 42. Body mass index | <18.5, ≥ 30 =1  25-<30 = 0.5  18.5-25 =0 | |  |
| 43. Handgrip strength | MALES:  For BMI ≤ 24, GS ≤ 29  For BMI 24.1–28, GS ≤ 30  For BMI >28, GS ≤ 32 =1 | FEMALES:  For BMI ≤ 23, GS ≤ 17  For BMI 23.1–26, GS ≤ 17.3  For BMI 26.1–29, GS ≤ 18  For BMI>29, GS ≤ 21 =1 |  |
| LAB VALUES | | | |
| 44. Glycohemoglobin (%) | 0-5.7 % =0, >5.7% =1 | |  |
| 45. Red blood cell count (million cells/μL) | M: 4.7-6.1= 0, Other =1 | F: 4.2-5.4=0, Other =1 |  |
| 46. Hemoglobin (g/dL) | M: 13.5-18= 0, Other=1 | F: 12-16 =0, Other = 1 |  |
| 47. Red cell distribution width (%) | 11.6-14.6 = 0, Other =1 | |  |
| 48. Lymphocyte percent (%) | 20- 40 = 0, Other=1 | |  |
| 49. Segmented neutrophils percent (%) | 40-80 = 0, Other=1 | |  |

**Table S2:** Baseline characteristics of participants according to frailty

| Characteristics | Total | Non-frail | Frail | P value |
| --- | --- | --- | --- | --- |
| Age,years | 61(53,71) | 59(51,69) | 63(55,73) | < 0.0001 |
| Gender (%) |  |  |  | < 0.001 |
| Female | 416(42.63) | 176(37.44) | 240(49.09) |  |
| Male | 735(57.37) | 399(62.56) | 336(50.91) |  |
| Race/ethnicity (%) |  |  |  | 0.45 |
| Mexican American | 56(1.21) | 28(1.17) | 28(1.26) |  |
| Non-Hispanic Black | 168(5.95) | 85(5.20) | 83(6.89) |  |
| Non-Hispanic White | 807(84.80) | 412(85.94) | 395(83.39) |  |
| Other Hispanic | 57(1.91) | 21(1.45) | 36(2.49) |  |
| Other Race - Including Multi-Racial | 63(6.12) | 29(6.24) | 34(5.98) |  |
| Marital status (%) |  |  |  | 0.01 |
| Married | 651(60.98) | 362(66.07) | 289(54.67) |  |
| Nonmarried | 500(39.02) | 213(33.93) | 287(45.33) |  |
| PIR (%) |  |  |  | < 0.0001 |
| < 1 | 271(17.22) | 96(9.58) | 175(26.71) |  |
| >= 1 | 880(82.78) | 479(90.42) | 401(73.29) |  |
| Educational level (%) |  |  |  | < 0.001 |
| College graduate or above | 104(10.36) | 74(14.44) | 30(5.28) |  |
| High school or below | 765(63.46) | 357(59.33) | 408(68.58) |  |
| Some college or associated degree | 282(26.19) | 144(26.22) | 138(26.14) |  |
| Smoking |  |  |  | 0.004 |
| Current | 480(42.96) | 227(40.50) | 253(46.01) |  |
| Former | 514(42.85) | 249(41.27) | 265(44.81) |  |
| Never | 157(14.19) | 99(18.24) | 58(9.18) |  |
| Drinking (%) |  |  |  | < 0.0001 |
| Former | 371(28.13) | 125(18.56) | 246(40.02) |  |
| Heavy | 187(17.69) | 113(21.44) | 74(13.03) |  |
| Mild | 383(33.52) | 207(35.76) | 176(30.74) |  |
| Moderate | 140(15.16) | 97(19.60) | 43(9.64) |  |
| Never | 70(5.49) | 33(4.64) | 37(6.56) |  |
| Energy intake(kcal/day) | 1851(1369,2475) | 2004(1508,2575) | 1671.00(1231,2340) | < 0.0001 |
| Protein intake(g/day) | 69.69(47.84,94.17) | 73.47(51.31,96.39) | 64.53(45.29,90.78) | 0.01 |
| **BRI** | **5.30(3.94,6.74)** | **4.97(3.60,6.08)** | **5.96(4.48,7.76)** | **< 0.0001** |
| BMI (kg.m2) | 27.00(23.70,31.63) | 26.39(23.50,30.10) | 28.10(23.90,34.00) | < 0.001 |
| Hypertension (%) |  |  |  | < 0.0001 |
| No | 444(43.29) | 289(55.44) | 155(28.20) |  |
| Yes | 707(56.71) | 286(44.56) | 421(71.80) |  |
| DM (%) |  |  |  | < 0.0001 |
| No | 851(78.48) | 493(88.85) | 358(65.60) |  |
| Yes | 300(21.52) | 82(11.15) | 218(34.40) |  |

*PIR* family income-to-poverty level ratio, *BRI* body roundness index, *BMI* body mass index, *DM* diabetes
